# Stigmatising attitudes towards people who inject drugs, and people living with blood borne viruses or sexually transmissible infections in a representative sample of the Australian population

**DOI:** 10.1371/journal.pone.0232218

**Published:** 2020-04-27

**Authors:** Timothy R. Broady, Loren Brener, Elena Cama, Max Hopwood, Carla Treloar

**Affiliations:** Centre for Social Research in Health, UNSW Sydney, Sydney, Australia; University of California San Diego, UNITED STATES

## Abstract

Stigma has significant detrimental health outcomes for those affected. This study examined socio-demographic characteristics that were associated with stigmatising attitudes among the general population towards people who inject drugs, and people living with blood borne viruses or sexually transmissible infections. Questions were included in the Australian Survey of Social Attitudes (total sample = 1,001). Attitudes towards each of the target populations were measured by 5-item stigma scales. Bivariate analyses and multiple regression analyses were conducted to identify socio-demographic characteristics associated with stigmatising attitudes. Knowing a person affected by a stigmatised attribute was associated with reduced stigmatising attitudes, while voting for a conservative political party was associated with increased stigmatising attitudes. Age, gender, education, income, and marital status were each related to some stigmatising attitudes. Results also highlight differences between attitudes towards a stigmatised behaviour (i.e., injecting drug use) and stigmatised conditions (i.e., blood borne viruses and sexually transmissible infections). Identifying socio-demographic characteristics that are associated with stigmatising attitudes may have global implications for informing stigma reduction interventions, in order to promote positive health outcomes for affected communities.

## Introduction

The negative consequences of health-related stigma have been well documented. In his seminal work, Goffman [1] defined stigma as an attribute which diverges from what is viewed as ‘normal’ by the majority and serves to discredit the stigmatised group. Stigma can have harmful effects on the psychological health, self-esteem, emotional well-being, and physical health of stigmatised groups [2–4], and there has been a growth of research on the implications of the enactment of stigma through discriminatory behaviours [5, 6].

Stigmatised attributes are viewed particularly negatively when they are associated with a threat of contagion (e.g., blood borne viruses [BBVs]), or when they are perceived to be within an individual’s control [7, 8]. For example, people who inject drugs (PWID), people living with BBVs (PLBBV), or people living with sexually transmissible infections (STIs) may be stigmatised because they are perceived to be responsible for their own behaviour and/or health conditions [9]. Consequently, PLBBV, people living with STIs (PLSTI), or PWID may be viewed as irresponsible and therefore be treated negatively due to fears that they will expose others to infection [9, 10]. Negative attitudes towards these groups are often informed by stereotypical representations in the media, such as those depicting PWID as unpredictable and volatile [10–12].

Stigma and its enactment via discriminatory behaviours in health care are associated with poorer health outcomes among stigmatised groups, including reduced engagement with health services, and poor treatment uptake and retention [5, 13]. In particular, negative attitudes held by health workers can impact the quality of care they provide to their clients, for example, by reducing their willingness to work with clients who possess stigmatised attributes (e.g., injecting drug use [IDU], BBVs, mental illness) [14, 15]. Further, negative attitudes can lead to health workers engaging in discriminatory practices, such as inappropriate infection control procedures, breaches of confidentiality, refusal to provide medical treatment, or rushed discharge from hospital [14, 16–18]. Conversely, supportive and non-judgemental health care settings can promote client engagement in health services, treatment retention, and ongoing health outcomes [19, 20].

While much research has focussed on the stigmatising attitudes held by health workers, less is known about how the general public views these stigmatised groups. Since seeking health care treatment is likely to necessitate disclosure of stigmatised behaviours or illnesses, affected individuals may choose to avoid disclosure in light of experiencing discrimination from broader society. Negative attitudes from the general public may therefore exacerbate any existing difficulties with negotiating health care for PWID, PLBBV, and those with STIs.

Previous research regarding mental health stigma has shown that attitudes towards stigmatised groups can be affected by specific socio-demographic characteristics, particularly age, gender and education [21, 22]. There are also a small number of studies that have examined factors associated with discriminatory attitudes towards PLHIV held by the general public. For example, Lim, Zelaya [23] found that socioeconomic factors at individual and community levels affected enacted stigma (i.e., discriminatory behaviour) towards PLHIV who inject drugs, with higher education in particular being associated with less discriminatory attitudes. Other studies have found that negative attitudes towards PLHIV are associated with being male, having completed lower formal education, and being older [24, 25].

Attitudes towards certain groups in society can also be affected by social and political conservatism. Conservatism generally aligns with right-wing political views, stressing traditional values and resistance to change [26]. This is often viewed in contrast to being socially progressive–that is, concerned with justice, equality, and tolerance. Research has identified that people at both ends of the political spectrum express prejudice towards those with different ideologies to their own [27]. Evidence generally suggests that conservatism is associated with prejudice towards and intolerance of a variety of social groups (e.g., ethnic minorities, LGBTI communities), while being socially progressive is associated with tolerance of typically marginalised groups [28, 29].

The characteristics typically associated with either conservatism or being socially progressive are often reflected in the policies and standpoints of various political parties. For example, in Australia, the Liberal/National Coalition is viewed as conservative, being most closely aligned with right-wing economics and an authoritarian social position. The Australian Greens are far less conservative (i.e., more economically left-wing and socially libertarian), and the Australian Labor party sits in between these two parties (for an overview, see the Political Compass: https://www.politicalcompass.org/aus2019). Recent research by Grimmer and Grube supports these characteristics [30]. They surveyed the Australian public on their perceptions of ‘brand attributes’ of major federal political parties. Respondents commonly associated the Coalition with terms such as ‘conservative’, ‘right wing’, and ‘economic management’, while the Labor party was commonly associated with terms including ‘unions’, ‘supports workers’, and ‘large spenders’, and the Greens were associated with terms such as ‘protecting the environment’, ‘social justice’, and ‘left wing’ [30].

While it is evident that certain socio-demographic variables may be associated with more negative attitudes, it is also important to assess factors which may reduce stigma and discrimination. As stigma and discrimination have a negative impact on health and wellbeing for people living with stigmatised health conditions, targeted interventions to reduce negative attitudes towards such groups remain a priority. Allport’s [31] seminal work argues that contact between social groups reduces prejudice. Supporting this theory, researchers have found that direct contact with people with a mental illness decreases the acceptance of stereotypes surrounding mental health [32, 33]. Similarly, contact with people living with either hepatitis C (HCV) or HIV has been shown to decrease negative perceptions about those infections [9, 34–36]. Despite this, there is limited evidence regarding the simultaneous associations between socio-demographic variables, contact with stigmatised groups, and attitudes towards those groups among the general public.

### Current study

This paper aims to investigate socio-demographic characteristics that are associated with stigmatising attitudes towards PLSTI, PLHIV, people living with HCV (PLHCV), and PWID within a representative sample of the Australian adult population. In addition, this study assesses whether personal contact with affected individuals moderates the relationship between socio-demographic variables and expressed stigma.

## Methods

This study was approved by the University of New South Wales Human Research Ethics Committee (approval no. HC16129).

This research utilised data collected in waves 2 to 4 of the 2017 Australian Survey of Social Attitudes (AuSSA), conducted between August 2017 and March 2018. The AuSSA is an annual postal survey, which collects data from a representative sample of the Australian adult population, randomly selected from the Australia Electoral Roll. Participants implied their consent by returning a completed survey. All data were fully anonymous. The theme of the 2017 AuSSA was ‘Social networks and social resources’.

The following socio-demographic variables are routinely collected through the AuSSA: gender (available responses were ‘male’ [coded 0] and ‘female’ [coded 1] only); age; highest completed educational qualification (0 = less that high school; 1 = high school; 2 = Certificate or Associate Degree; 3 = Bachelor or Graduate Diploma; 4 = Masters or Doctorate); employment status (0 = not employed; 1 = employed); country of birth (0 = Australia; 1 = overseas); Aboriginal or Torres Strait Islander (ATSI) (0 = no; 1 = yes); monthly household income (in Australian dollars; 0 = less than $3,500; 1 = $3,500-$6,499; 2 = $6,500-$10,999; 3 = $11,000 or more); area of residence (0 = big city, 1 = town/small city, 2 = country); and marital status (married, never married, previously married [including separated, divorced, and widowed]). Marital status was dummy coded into two separate variables–married (0 = not currently married; 1 = currently married) and ever married (0 = never married; 1 = ever married).

Detailed measures of political conservatism were not included in the AuSSA, therefore, recent voting history (i.e., at the most recent Australian federal election) was included in analyses as a proxy measure of conservatism. In line with the public perceptions of the major parties reported by Grimmer and Grube [30], voting for the Coalition was considered to be representative of the most conservative views, voting for the Greens was considered to represent the least conservative views, and voting for the Labor party was considered to be in between these two. These variables were each dummy coded as 1 = voted for the party and 0 = did not vote for the party.

A series of five-item stigma scales related to PLHIV, PLHCV, PWID, and PLSTI were included in the 2017 AuSSA. These scales were adaptations of scales previously used in relation to PWID and PLHCV [19]. Items reflected stigmatising attitudes towards PWID, PLHIV, PLHCV, and PLSTI (e.g., ‘I can tell by looking at someone if s/he has hepatitis C’, ‘I could not be friends with someone who has HIV’, ‘People who are infected with a STI deserve what they get’). Participants responded on a 5-point scale from 1 (strongly agree) to 5 (strongly disagree). Positively worded items (e.g., ‘People should feel sympathetic and understanding of injecting drug users’) were reverse scored and scale items were summed to produce scores of PLHIV stigma (α = .84), PLHCV stigma (α = .62), PLSTI stigma (α = .81), and PWID stigma (α = .72), each with a possible range of 5–25, where higher scores reflected more stigmatising attitudes. As a measure of personal contact, participants were asked if they personally knew someone who injects drugs, someone who has HIV, someone who has HCV, or someone who has had an STI (response options were 1 = yes or 0 = no).

Comparisons of mean stigma scores between socio-demographic groups were conducted using independent t-tests and one-way ANOVAs with post-hoc Bonferroni comparisons. Following these bivariate analyses, multivariable analyses were conducted to determine which variables retained an independent effect on stigma scale scores. Due to its relatively low scale reliability, multivariable analyses were not conducted with HCV stigma scale scores. Socio-demographic variables that were significantly associated with stigma scores in the bivariate analyses (*p* < .05 level) were included as independent variables in multiple regression models with stigma scores as the dependent variable. Interaction terms were also created between each of the socio-demographic variables and knowing a person with HIV, who has had an STI, or who injects drugs to determine if knowing a person with these attributes moderated the relationship between participants’ demographic characteristics and stigma.

## Results

The final sample consisted of 1,001 adult Australians from the three AuSSA waves. A summary of demographic characteristics of the full sample is shown in Table 1.

**Table 1:** Participants’ socio-demographic characteristics.

N = 1,001	N (%)^1^
**Gender:**	
Female	554 (56.5)
Male	427 (43.5)
**Age:** M (SD), Range	54.34 (7.09), 18–93
<30 years	102 (10.8)
30–49 years	237 (25.0)
50–69 years	415 (43.8)
70+ years	194 (20.5)
**Highest education:**	
Less high school	184 (18.9)
High school	104 (10.7)
Certificate–Associate Degree	355 (36.5)
Bachelor–Graduate Diploma	256 (26.3)
Masters–Doctorate	73 (7.5)
**Employment status**	
Currently employed	566 (56.5)
Not employed	435 (43.5)
**Country of birth**	
Australia	739 (75.9)
Overseas	235 (24.1)
**Aboriginal or Torres Strait Islander**	
Yes	23 (2.4)
No	945 (97.6)
**Monthly household income:**	
<AU$3500	171 (24.4)
AU$3500–6499	181 (25.8)
AU$6500–10999	167 (23.8)
AU$11000+	183 (26.1)
**Area of residence:**	
Big city	592 (61.3)
Town/small city	233 (24.1)
Country	141 (14.6)
**Marital status:**	
Married	579 (60.2)
Previously married	174 (18.1)
Never married	209 (21.7)
**Party voted for at last election:**	
Liberal/National	399 (45.7)
Labor	306 (35.1)
Greens	109 (12.5)
Other party	59 (6.8)
**Personally know:**^2^	
PWID	97 (10.1)
PLHIV	57 (6.1)
PLHCV	92 (9.8)
PWSTI	171 (18.2)

^1^% reflects the valid percent of each variable (missing data have been excluded)

^2^ Items were not mutually exclusive.

Table 2 shows comparisons between socio-demographic groups (nominal variables) on each of the stigma scales, and Table 3 shows correlations between socio-demographic variables (ordinal and scale variables) and scores on each of the stigma scales.

**Table 2:** Comparison of mean people who inject drugs, people living with HIV, people living with hepatitis C, and people living with sexually transmissible infections stigma scale scores between socio-demographic groups.

	PWID Stigma M (SD)	PLHIV Stigma M (SD)	PLHCV Stigma M (SD)	PLSTI Stigma M (SD)
**Full sample**	17.94 (3.69)	11.54 (3.77)	12.81 (2.52)	11.88 (3.69)
**Gender:**				
Female	17.88 (3.57)	11.03 (3.57)	12.54 (2.43)	11.41 (3.58)
Male	18.00 (3.80)	12.19 (3.91)	13.15 (2.60)	12.46 (3.75)
	*t*_(943)_ = 0.46, *p* = 0.64	*t*_(943)_ = 4.74, *p<*0.001	*t*_(941)_ = 3.71, *p<*0.001	*t*_(944)_ = 4.41, *p*<0.001
**Employment status**				
Currently employed	17.66 (3.72)	11.02 (3.69)	12.49 (2.42)	11.17 (3.54)
Not employed	18.34 (3.61)	12.27 (3.75)	13.25 (2.60)	12.86 (3.68)
	*t*_(952)_ = 2.82, *p* = 0.01	*t*_(953)_ = 5.16, *p<*0.01	*t*_(950)_ = 4.61, *p<*0.001	*t*_(953)_ = 7.18, *p*<0.001
**Country of birth**				
Australia	17.91 (3.72)	11.23 (3.64)	12.72 (2.50)	11.61 (3.57)
Overseas	17.94 (3.57)	12.40 (3.99)	13.18 (2.48)	12.59 (3.94)
	*t*_(938)_ = 0.09, *p* = 0.93	*t*_(940)_ = 4.14, *p*<0.001	*t*_(937)_ = 2.40, *p* = 0.02	*t*_(941)_ = 3.52, *p*<0.001
**Area of residence:**				
Big city	17.85 (3.74)	11.51 (3.77)	12.71 (2.58)	11.84 (3.83)
Town/small city	18.08 (3.49)	11.35 (3.79)	12.84 (2.42)	11.67 (3.54)
Country	18.17 (3.69)	11.84 (3.73)	13.21 (2.39)	12.27 (3.34)
	*F*_(2,926)_ = 0.61, *p* = 0.55	*F*_(2,928)_ = 0.71, *p* = 0.49	*F*_(2,925)_ = 2.30, *p* = 0.10	*F*_(2,929)_ = 1.17, *p* = 0.31
**Marital status:**				
Married	18.37 (3.60)	11.84 (3.66)	12.87 (2.51)	12.14 (3.67)
Previously married	17.64 (3.47)	11.68 (3.60)	13.10 (2.55)	12.15 (3.46)
Never married	17.07 (3.88)	10.57 (4.03)	12.46 (2.49)	10.94 (3.80)
	*F*_(2,921)_ = 10.17, *p*<0.001	*F*_(2,923)_ = 8.81, *p*<0.001	*F*_(2,920)_ = 3.19, *p* = 0.04	*F*_(2,924)_ = 8.55, *p*<0.001
**Know affected person/s**				
Yes	16.66 (4.62)	7.96 (2.29)	12.23 (2.28)	9.67 (3.39)
No	18.12 (3.54)	11.78 (3.70)	12.89 (2.53)	12.35 (3.57)
	*t*_(932)_ = 3.68, *p*<0.001	*t*_(920)_ = 7.66, *p*<0.001	*t*_(918)_ = 2.38, *p* = 0.02	*t*_(922)_ = 8.96, *p*<0.001

**Table 3:** Correlations between socio-demographic variables and people who inject drugs, people living with HIV, people living with hepatitis C, and people living with sexually transmissible infections stigma scale scores.

	PWID Stigma	PLHIV Stigma	PLHCV Stigma	PLSTI Stigma
**Age**	*r* = .10, *p* = .002	*r* = .21, *p* < .001	*r* = .17, *p* < .001	*r* = .26, *p* < .001
**Education**	*ρ* = -.13, *p* < .001	*ρ* = -.15, *p* < .001	*ρ* = -.17, *p* < .001	*ρ* = -.16, *p* < .001
**Household income**	*r* = -.003, *p* = .95	*r* = -.08, *p* = .03	*r* = -.03, *p* = .37	*r* = -.12, *p* = .002
**Conservatism**	*ρ* = .32, *p* < .001	*ρ* = .27, *p* < .001	*ρ* = .25, *p* < .001	*ρ* = .25, *p* < .001

Scores on the PWID stigma scale (M = 17.94, SD = 3.69) were considerably higher than scores on the PLHIV stigma scale (M = 11.54, SD = 3.77), the PLSTI stigma scale (M = 11.88, SD = 3.69), and the PLHCV stigma scale (M = 12.81, SD = 2.52). This suggests that participants held greater negative attitudes towards PWID, compared to the other groups. Males reported higher PLHIV, PLHCV, and PLSTI stigma scores than females, though there was no gender difference in PWID stigma scores. Participants born overseas reported higher PLHIV, PLHCV, and PLSTI stigma scores, and those with lower household incomes reported higher PLHIV and PLSTI stigma scores. Higher scores on each of the stigma scales were associated with older participant age, lower levels of formal education, not being employed, and being married (currently or previously). Scores on each of the stigma scales were lowest among those who voted for the Greens at the last federal election (i.e., the most progressive), and highest among those who voted for the Coalition (i.e., the most conservative). Stigma scores were not associated with area of residence. For each of the four attributes, participants who knew someone living with the attribute reported lower stigma scores than those who did not know anyone affected.

### People living with HIV stigma

The final multiple regression model with PLHIV stigma as the dependent variable is shown in Table 4.

**Table 4:** Multiple linear regression results with people living with HIV stigma scale scores as the dependent variable.

Variable	*Unstandardised B*	S.E.	*Standardised β*	*p*
Constant	11.65	.78		< .001
Know PLHIV	-3.23	.50	-.23	< .001
Gender	.47	.27	-.07	.08
Age	.02	.01	.11	.04
Education	-.23	.12	-.07	.06
Employed	-.03	.32	-.004	.92
Born overseas	.87	.31	.10	.01
Income	-.27	.13	-.08	.04
Ever married	.43	.39	.05	.26
Voted for Coalition	-.17	.41	-.02	.67
Voted for Labor	-1.41	.43	-.18	.001
Voted for Greens	-2.57	.51	-.25	< .001
		Adj. *r*^*2*^ = .20		

Less negative attitudes towards people living with PLHIV (as measured via the PLHIV stigma scale) were associated with knowing someone with HIV, being younger, being born in Australia, higher household income, and voting for either the Labor party or the Greens party at the last election. Knowing someone with HIV did not form an interaction effect with any other variables.

### People living with sexually transmissible infections stigma

The final multiple regression model with PLSTI stigma as the dependent variable is shown in Table 5.

**Table 5:** Multiple regression results with people living with sexually transmissible infections stigma scale scores as the dependent variable.

Variable	*Unstandardised B*	S.E.	*Standardised β*	*p*
Constant	12.34	.77		< .001
Know PLSTI	-1.46	.33	-.17	< .001
Gender	-.48	.26	-.07	.06
Age	.02	.01	.11	.03
Education	-.13	.12	-.04	.29
Employed	-.14	.32	-.02	.65
Born overseas	.58	.30	.07	.06
Income	-.44	.13	-.14	.001
Ever married	.09	.37	.01	.82
Voted for Coalition	-.10	.40	-.01	.81
Voted for Labor	-1.09	.42	-.14	.01
Voted for Greens	-2.57	.50	-.25	< .001
		Adj. *r*^*2*^ = .19		

Less negative attitudes towards PLSTI (as measured via the PLSTI stigma scale) were associated with knowing a person who has had an STI, being younger, higher household income, and voting for either the Labor party or the Greens party at the last election. Knowing a person who has had an STI did not form an interaction effect with any other variables.

### People who inject drugs stigma

The final multiple regression model with PWID stigma as the dependent variable is shown in Table 6.

**Table 6:** Multiple regression results with people who inject drugs stigma scale scores as the dependent variable.

Variable	*Unstandardised B*	S.E.	*Standardised β*	*p*
Constant	18.34	.63		< .001
Know PWID	-.17	.53	-.01	.76
Age	.001	.01	.01	.89
Education	-.35	.10	-.11	.001
Employed	-.10	.28	-.01	.72
Married	.68	.25	.09	.01
Voted for Coalition	.87	.34	.12	.01
Voted for Labor	-.24	.37	-.03	.52
Voted for Greens	-2.21	.48	-.19	< .001
Voted for Labor x Know PWID	-2.05	.89	-.10	.02
Voted for Greens x Know PWID	-3.14	1.19	-.10	.01
		Adj. *r*^*2*^ = .14		

**Fig 1:**
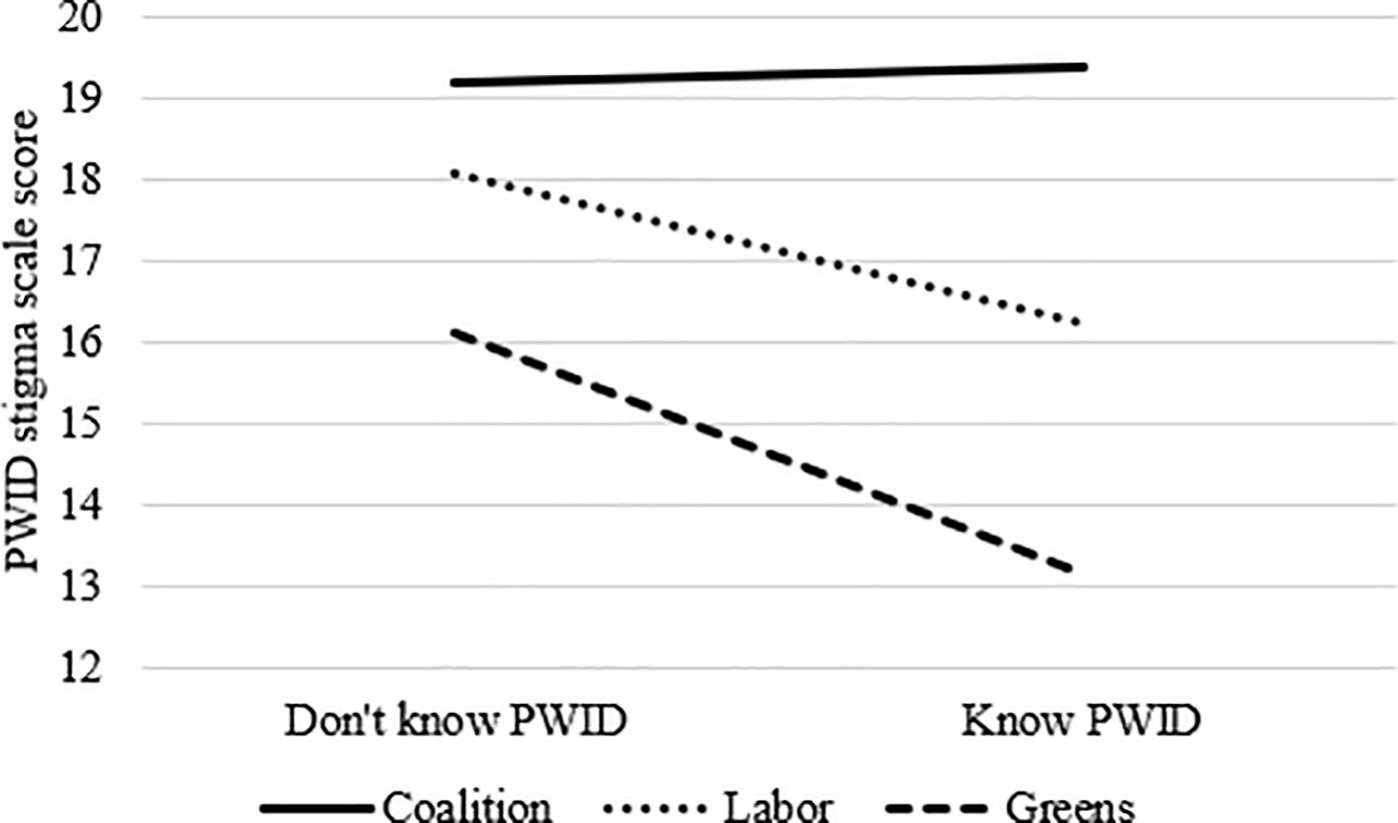
Interaction effect between political conservativism and knowing people who inject drugs.

Less negative attitudes towards PWID (as measured via the PWID stigma scale) were associated with higher education levels, not being married, and voting for the Greens party at the last election. Voting for the Coalition at the last election was associated with holding more negative attitudes towards PWID. There were also interaction effects between knowing a person who inject drugs and voting for the Labor party or the Greens party. Knowing PWID was not directly associated with stigma scores but was associated with lower stigma among those who voted for either of those parties at the last election (see Fig 1).

## Discussion

This paper aimed to identify socio-demographic characteristics that are associated with holding stigmatising attitudes towards PWID, PLBBVs, and PLSTI, and whether personal contact with affected individuals and voting history moderated those relationships. In multivariable analyses, more stigmatising attitudes were evident among participants who were older and those with lower household incomes (in relation to PLHIV and PLSTI), those who had completed lower levels of education and who were not married (in relation to PWID), and those who were born overseas (in relation to PLHIV). Voting for either of the two less conservative political parties (i.e., Labor or the Greens) was associated with less stigmatising attitudes towards PLHIV and PLSTI. Voting for the Greens was also independently associated with less stigmatising attitudes towards PWID, while voting for the Coalition (i.e., the most conservative of the three parties) was independently associated with more stigmatising attitudes towards PWID.

As has been shown in previous research (e.g., 34, 36), personal contact with affected individuals was associated with less stigmatising attitudes towards PLHIV and PLSTI, however, contact was not independently associated with stigma towards PWID. Interaction effects were evident, however, whereby knowing PWID was associated with lower levels of IDU-related stigma among those who voted for the Greens or the Labor party, but not among those who voted conservatively (i.e., for the Coalition).

Further research is warranted to investigate the effects of conservatism on stigmatising attitudes and subsequent behaviour. Additionally, increased understandings of various aspects of political, cultural, and economic conservatism in relation to the stigmatised groups identified in this study may assist in developing and targeting any interventions to reduce their experiences of stigma. Previous research has linked culturally conservative attitudes with resistance to change, dogmatism and a desire for certainty and security, however, economic conservatism was not found to be associated with rigid thinking or aggression towards others with opposing points of view [37, 38]. It may therefore be more difficult to change any stigmatising attitudes held by those with more culturally (but not necessarily economic) conservative views.

It is important to highlight that while participants with a more conservative voting history reported more stigmatising attitudes, negative attitudes were apparent to some degree across all voting groups. Unlike with PLHIV/PLSTI, increasing contact with PWID may not be an effective method of reducing stigmatising attitudes across the general population as a whole without additional complementary intervention strategies. In fact, contact may reinforce negative attitudes if it is not a positive experience for each group [31] Complementary strategies may include highlighting social, cultural, and economic implications arising from stigma throughout society, while ensuring that targeted interventions are long-term in nature. Evidence regarding the long-term effects of short-term stigma reduction interventions is inconclusive [39–41]. Coupled with the potential difficulty associated with shifting politically and culturally conservative views, there is a clear need for extensive investment in large-scale and long-term intervention efforts.

The independent effect of knowing a person with HIV/STIs on stigma scores suggests that familiarity with affected people and putting a human face to these infections may contribute to a shift in stigmatising attitudes, even among those groups within the general population who are most likely to hold these discriminatory views. Previous research with health care professionals has found that listening to people who live with hepatitis C or HIV speaking publicly about their illness helps to deconstruct stereotypes and increase positive attitudes among audience members [34–36]. This approach may therefore have similar effects in relation to other stigmatised attributes and with broader audiences, including the general public.

These results highlight similarities in the general public’s attitudes towards PLHIV and PLSTI, while also identifying how attitudes towards PWID may differ. An important finding was that attitudes towards PWID were more negative than towards the other groups. In addition, the socio-demographic characteristics associated with stigma towards PWID were different to those associated with stigma towards PLHIV and PLSTI. Specifically, being older, having a lower income, voting for a more conservative political party, and not knowing someone with HIV/STIs were all directly associated with higher levels of reported stigmatising attitudes towards people with HIV/STIs.

Identifying characteristics of those with the most stigmatising attitudes towards PWID was less straightforward. Unlike PLHIV/PLSTI stigma, age and income were not associated with PWID stigma in the multiple regression analysis, however, PWID stigma was higher than both PLHIV and PLSTI stigma across all age groups and income categories. Lower levels of formal education, and not being married were associated with higher PWID stigma scores. This is in line with existing literature that has found socioeconomic status and inequality to be associated with enacted stigma towards PLHIV and PWID [23]. It is also of note that interactions between knowing PWID and recent voting history were associated with PWID stigma scores, though no comparative interactions were evident in relation to HIV or STIs. This further highlights the differences between public perceptions of PWID and those of PLHIV/PLSTI.

These differences highlight differences in public perceptions of stigmatised behaviours (i.e., IDU) and stigmatised conditions (i.e., HIV, HCV, STIs) and may be due to perceptions that IDU is a personal choice and is under the control of PWID. As previously noted, when people believe that a stigmatised attribute is under the control of the individual, they are more likely to discriminate based on that attribute [7–9]. Results from this study suggest that an individual’s social or political view can either reinforce or mitigate such beliefs, attitudes, and behaviours. The potential for intersecting stigmas among PWID must also be noted. Members of the general public may be more likely to hold stigmatising attitudes towards PWID, not just as a result of their injecting drug use, but also due to assumptions that they have, or are at risk of acquiring BBVs, or that they are likely to behave dangerously towards others [18, 19, 23]. Any attempts to reduce stigmatising attitudes within the general population must account for the potential overlap between different stigmatised behaviours and conditions, and how these may simultaneously manifest.

The importance of addressing stigmatising or discriminatory views among the general public can be seen in the effects that public opinion and/or media attention can have on public health services. For example, negative views towards PWID held by vocal groups within the general population can translate into value-laden media reporting, which can in turn influence decisions to close public health facilities such as needle and syringe programs [42].

It is worth noting the relatively low proportions of participants reporting that they knew PLHIV (6.1%), PLHCV (9.8%), PWID (10.1%), or PLSTIs (18.2%). More participants may have personally known people within these groups, but had not been aware of it due to stigmatised individuals concealing their stigmatised attribute rather than face potential negative treatment from others [6]. Considering the low levels of personal interactions with these individuals, it may be that the vast majority of the Australian population have limited understanding of the realities of life for these affected communities. Further, due to their lack of interaction, many individuals may not see the value or necessity in them being more educated about issues facing PWID, or those living with BBVs/STIs. An ongoing public health challenge is to break down socio-demographic barriers that preclude members of the general population from being exposed to and consequently understanding the experiences of these stigmatised groups. Directly targeted advocacy interventions can result in more positive attitudes towards services for PWID [43], though the design and wording used can have important implications [44]. In the context of mental health, social contact has been shown to be the most effective mechanism for reducing stigmatising attitudes, though long-term evidence is lacking. Furthermore, evidence regarding the effectiveness of large scale media or social media campaigns is similarly lacking, and analysis of potential unintended negative consequences of such approaches has not been undertaken [39].

### Limitations

This study is limited by its cross-sectional nature. Causality between socio-demographic variables and stigmatising attitudes cannot be assumed, nor can the available data demonstrate any relationship between participants’ attitudes and any resultant behaviour. Behaviour may be directed more by implicit or subconscious attitudes than by explicit, self-reported attitudes [45]. The reliability of the PLHCV stigma scale was insufficient to conduct multivariable analyses, however, bivariate analyses demonstrated similar results to the PLHIV and PLSTI stigma scales. Developing a more reliable scale of PLHCV stigma would be beneficial for future research in this field. Using recent voting history as a proxy for political ideology is also a limitation. Given the significant effects of this proxy measure in relation to stigma in this study, further research is warranted to more fully investigate the relationship between political conservatism and stigma towards these affected groups (e.g., using validated scales).

### Conclusions

The socio-demographic variables included as independent variables in the regression models do not represent opportunities for interventions to reduce stigma (e.g., it is difficult to change demographic variables such as income, age, or education level). However, identifying socio-demographic characteristics that are associated with stigmatising attitudes can inform targeted intervention efforts to reduce stigma at structural, community, and individual levels. Reducing negative attitudes towards stigmatised groups remains a public health imperative, in order to promote the health and wellbeing of affected communities.
